# Hepatoprotective Effect of Superfine Particles of Herbal Medicine against CCl_4_-Induced Acute Liver Damage in Rats

**DOI:** 10.1155/2014/934732

**Published:** 2014-06-09

**Authors:** Gang Cao, Qinglin Li, Xiaocheng Chen, Hao Cai, Sicong Tu

**Affiliations:** ^1^Research Center of TCM Processing Technology, Zhejiang Chinese Medical University, Hangzhou 310053, China; ^2^Zhejiang Cancer Hospital, Hangzhou, China; ^3^The First Affiliated Hospital, Wenzhou Medical University, Wenzhou, China; ^4^Department of Chinese Materia Medica, College of Pharmacy, Nanjing University of Chinese Medicine, Nanjing 210023, China; ^5^Faculty of Medicine, University of New South Wales, Sydney, NSW 2031, Australia

## Abstract

This study aimed to examine the hepatoprotective effects of the superfine particles of *Radix Tetrastigma* (SPRT) against CCl_4_-induced acute liver damage in rats. Animals were treated with SPRT (0.3, 0.6, and 1.2 g/kg) and showed remarkable hepatoprotection against CCl_4_-induced hepatotoxicity. CCl_4_ altered various biochemical parameters in rat liver, including alanine aminotransferase (ALT), aspartate aminotransferase (AST), malondialdehyde (MDA), superoxide dismutase (SOD), histopathological changes, and Bax and caspase-3 expressions. SPRT significantly prevented increases in ALT and AST levels, reduced MDA level, decreased Bax and caspase-3 protein expression, enhanced SOD activity, and provided significant amelioration in the histopathological lesions. These findings suggested that SPRT has significant protective effect against acute hepatotoxicity induced by CCl_4_ in rats.

## 1. Introduction

Herbal medicine has been applied for disease prevention and treatment for thousands of years [1, 2]. Herbal medicine can boost immunity and protect the liver, regulate liver function, improve blood circulation in the liver, effectively repair liver cell damage, and relieve symptoms of liver diseases [3–5].

*Radix Tetrastigma*, also called San ye-qing, one of the oldest tonic herbal medicines, has attracted increasing attention as one of the most popular and valuable herbal medicines in clinical application.* Radix Tetrastigma* can be used as medicine, healthy food, and cosmetics because of its biological and pharmacological activities, including anti-inflammation, antivirus, antioxidation, heat-removing, detoxification, antihepatotoxicity, and analgesia [6, 7]. The root of* Radix Tetrastigma* contains various flavonoids, such as rutin, quercetin, and kaempferol [8, 9].

*Radix Tetrastigma* is a rare plant and could not be cultured. Unfortunately, its natural supply is now significantly decreasing. Traditional processing procedures of* Radix Tetrastigma* include section baking and drying, sulfur fumigation, and storage in a woven bag. The disadvantages of these procedures include high water content, mold growth, infestation, and unsuitability for long-term storage. More importantly, the essences can be lost during the baking process and the residual sulfur dioxide produced during sulfur fumigation can be harmful to humans. Traditional decoction of* Radix Tetrastigma* can achieve efficacy of only 3% to 5%, resulting in the loss of 95% of the bioactive components. Even when 3% to 5% of bioactive components are obtained from water, the decoction tends to be volatilized. Evidently, the traditional processing technique is not efficient. Thus, improving the processing technique is urgently needed to avoid unnecessary loss of precious* Radix Tetrastigma*. This study was conducted to develop a superfine processing technique using superfine particles to process* Radix Tetrastigma*. The proposed technique allows active components to be absorbed without relying on their active transportation through the cell membrane. Superfine particles present large surface areas to interact with target molecules. When these particles are conjugated with medicine, the conjugates offer higher surface charges and surface adhesion, resulting in higher efficacies and better medicine absorption. The efficacy and medicine absorption could be improved by more than 90%, and the medicinal dosage can be saved by more than 90%. This proposed technique can efficiently preserve the bioactive constituents in the original medicine, accelerate the release of bioactive constituents, and improve their bioavailability [10, 11].

Previous studies have reported that polysaccharides in* Radix Tetrastigma* have strong antioxidant and antihepatotoxic activities [12, 13]. However, information on the hepatoprotective effect of the superfine herbal particles of* Radix Tetrastigma in vivo* is limited. This study was undertaken to evaluate the protective effect of the superfine particles of* Radix Tetrastigma* (SPRT) against CCl_4_-induced hepatotoxicity.

## 2. Materials and Methods

### 2.1. Preparation of SPRT

*Radix Tetrastigma* was obtained from Henan suppliers and authenticated by an expert in the field. SPRT was prepared by using the mechanical ball milling technique in the Research Center of TCM Processing Technology, Zhejiang Chinese Medical University. The power particle size was less than 13 *μ*m, and the recovery rate was more than 98%.

### 2.2. Reagents

Carbon tetrachloride (CCl_4_) was purchased from Guangdong Dajinhua Chemical Co., Ltd. (Guangdong, China). Alanine aminotransferase (ALT) and aspartate aminotransferase (AST) assay kits were purchased from Ningbo Meikang Biotech Co., Ltd. (Zhejiang, China). Superoxide dismutase (SOD) and malondialdehyde (MDA) assay kits were purchased from Jiancheng Biotech Co., Ltd. (Nanjing, China). Bax and caspase-3 antibodies were purchased from Wuhan Boshide Biotechnology Co., Ltd. (Wuhan, China). All other chemicals were purchased from Nanjing Ronghua Reagent Co. (Nanjing, China).

### 2.3. Animals

Adult male imprinting control region rats (20 ± 2 g body weight) were purchased from the Laboratory Animal Center of Zhejiang Academy of Medical Sciences (Zhejiang, China) and maintained at the Experimental Animal Center of Zhejiang Chinese Medical University. Animals were acclimatized for at least five days with alternating 12 h dark/light cycles in a climate-controlled room where the temperature was maintained at 25 ± 2°C and relative humidity of 60% ± 10%. Water and standard laboratory food were made available ad libitum. All experiments were performed according to the guidelines for the care and use of animals as established by Zhejiang University.

### 2.4. Experimental Design

Hepatotoxicity was orally induced with 0.1% of CCl_4_ (20 mL/kg body weight in peanut oil). SPRT (0.3, 0.6, and 1.2 g/kg) and the standard hepatoprotective drug, bifendate, were prepared in 1% sodium carboxymethyl-cellulose (CMC). Rats were randomly divided into six groups (10 rats/group). The rats in group A served as normal controls and were only treated* per orem* (p.o.) with the vehicle (20 mL/kg of 1% CMC-Na) daily for seven days. The rats in group B served as the disease models and received intraperitoneal injection with 0.1% of CCl_4_ (0.1 : 100 of CCl_4_ in peanut oil, 20 mL peanut oil/kg body weight) on the seventh days and using a vehicle for the rest of the days. The rats in groups C, D, and E were given SPRT p.o. at 0.3, 0.6, and 1.2 g/kg body weight for seven days, respectively, and 20 mL/kg o.p. of 0.1% CCl_4_ on the seventh day.

All rats were sacrificed 16 h after the final treatment. Blood was collected and serum was separated to evaluate the biochemical markers of hepatic injury. Liver was harvested for biochemical and histopathological studies as described previously.

### 2.5. Histopathology

A portion of the right lobe of the liver was preserved in 10% formalin solution and cut into 5 *μ*m sections and stained with hematoxylin and eosin (HE) for microscopic observation.

### 2.6. Immunohistochemistry

Immunoperoxidase staining was performed on formalin-fixed and paraffin-embedded 5 *μ*m tissue sections. Sections were fixed on microscope slides. The microscope slides were treated with poly-L-lysine to prevent capping. Microwave antigen retrieval was performed. The expressions of Bax and caspase-3 were quantitatively evaluated using a digital microscope equipped with a computer-aided image analysis system. The intensity index was determined using average integral optical density (AIOD).

### 2.7. Statistical Analysis

Data were presented as mean ± SD of ten animals each group. Statistical significance was determined using one-way analysis of variance. If the variances were unequal, LSD's multiple range tests and rank-sum tests were used. *P* value of <0.05 was considered statistically significant.

## 3. Results and Discussion

### 3.1. Effect of SPRT on Serum Marker Enzymes

**Figure 1:**
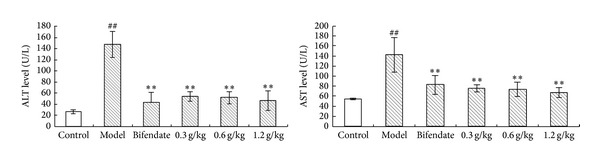
Effects of SPRT on biochemical parameters in CCl_4_-induced hepatotoxicity in rats. Data are expressed as mean ± SD. ^##^ *P* < 0.01, versus normal controls. **P < 0.01, versus disease model group.

The effects of SPRT on serum marker enzymes are presented in Figure 1. CCl_4_ increased the enzymatic activity of AST and ALT compared with the control group, indicating liver damage. Administration of SPRT at the doses of 0.3, 0.6, and 1.2 g/kg remarkably prevented hepatotoxicity induced by CCl_4_ in a dose-dependent manner.

### 3.2. Effect of SPRT on Liver MDA and SOD Activity

**Figure 2:**
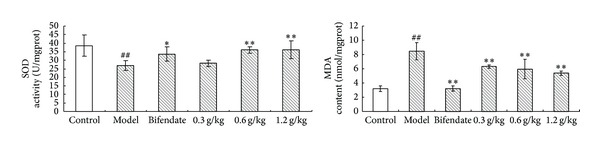
Effects of SPRT on antioxidant profile of CCl_4_-induced hepatotoxicity in rats. Data are expressed as mean ± SD. ^##^ *P* < 0.01, versus normal controls. **P < 0.01, versus disease model group. *P < 0.05, versus disease model group.

CCl_4_ treatment significantly increased the level of MDA in liver tissue compared with the control group. Meanwhile, CCl_4_ decreased the activity of hepatic antioxidant enzyme SOD compared with the respective control group. Treatment with SPRT at doses of 0.3, 0.6, and 1.2 g/kg significantly reduced the MDA level compared with the CCl_4_-treated rats. The liver of the animal treated with 0.6 and 1.2 g/kg of SPRT showed significant increase in SOD levels compared with the CCl_4_-treated rats. The results are shown in Figure 2.

### 3.3. Histological Analysis

**Figure 3:**
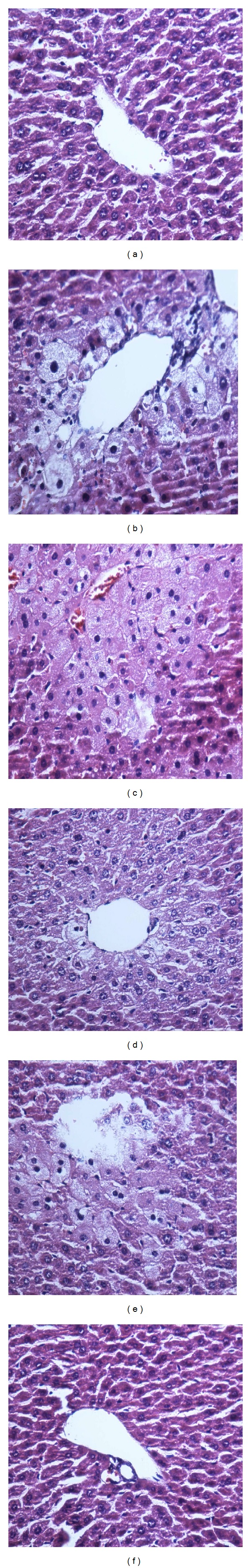
Effects of SPRT on the histopathological changes in liver of rats treated with CCl_4_. (a) Normal controls; (b) disease model group; (c) bifendate + 0.1% of CCl_4_; (d) SPRT (0.3 g/kg) and 0.1% of CCl_4_ group; (e) SPRT (0.6 g/kg) and 0.1% of CCl_4_ group; (f) SPRT (1.2 g/kg) and 0.1% of CCl_4_ group. HE staining; original magnification ×400.

Figure 3 showed the well-formed hepatocytes in an intact hepatic lobule of normal rat liver. The liver sections of CCl_4_-treated rats showed massive vacuolization, fatty changes, ballooning degeneration, and inflammatory cell infiltration, especially in the periportal hepatocytes. The histopathological hepatic lesions were significantly improved by the preadministration of SPRT or bifendate compared with the disease model group. These results indicate the hepatoprotective potential of SPRT.

### 3.4. Bax and Caspase-3 Expression in Liver

**Figure 4:**
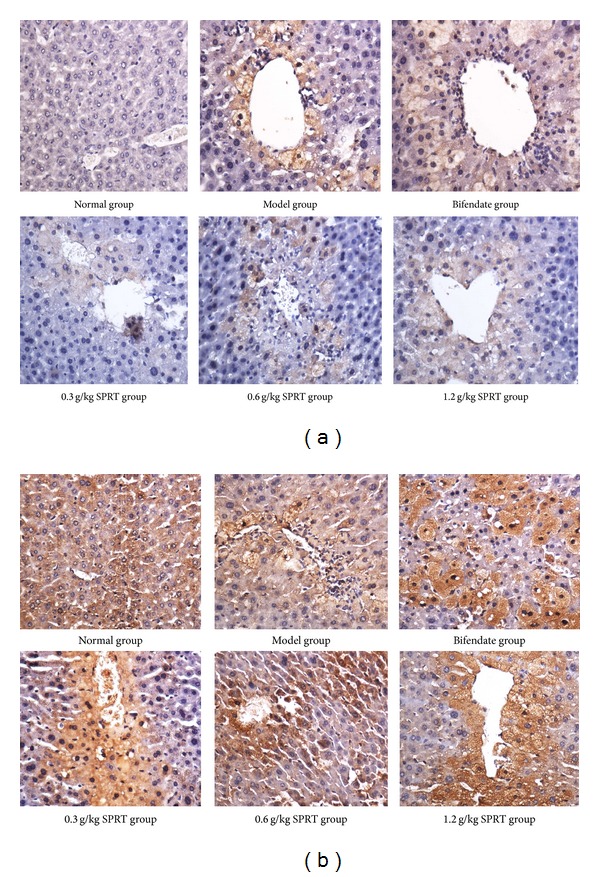
Effects of SPRT on the Bax (a) and caspase-3 (b) expression in rat liver treated with CCl_4_.

CCl_4_ treatment caused a significant increase in Bax and caspase-3 in the liver tissue. SPRT or bifendate treatment significantly inhibited both Bax and caspase-3 expressions (Figure 4).  Table 1 shows that Bax and caspase-3 in the disease model group were higher than those in normal control group (*P* < 0.01). Compared with the disease model group, the Bax and caspase-3 ICH indexes were significantly lower in the two doses of SPRT-treated group (0.6 and 1.2 g/kg; *P* < 0.05). No significant difference was observed between the disease model group and SPRT treatment group at 0.3 g/kg.

Hepatotoxicity induced by CCl_4_ is a classic disease model used for evaluation of hepatoprotective activity of herbal medicines. This study showed a significant increase in the activities of AST and ALT in serum after CCl_4_ treatment. These changes by preadministration of SPRT are reversible, implying that SPRT prevents liver damage.

SOD is a cellular antioxidant with a strong capability to remove superoxide anion and inhibit lipid peroxidation in which MDA is the main product. CCl_4_ treatment increased the SOD levels in the liver, leading to a low MDA level. The reduced activity of SOD and increased MDA level indicated hepatic damage in rats, which was reversed by the preadministration of SPRT at 0.3, 0.6, and 1.2 g/kg, suggesting the antioxidation effect of SPRT.

Hepatocyte apoptosis is a complex process regulated by multiple genes. Positive immunohistochemical staining of Bax and caspase-3 confirmed liver cell apoptosis induced by CCl_4_. The positive expression of Bax and caspase-3 decreased with increasing doses of SPRT treatment, suggesting that hepatocyte apoptosis was reversed by SPRT. This finding agreed with those of other studies [14].

Based on our experimental findings, SPRT has a protective effect against hepatotoxic CCl_4_. The hepatoprotective effects of SPRT are likely related to the free radical scavenging effect, which increases antioxidant activity, against membrane lipid peroxidation, and protects membrane integrity and function of liver cells. Further studies are needed to investigate the molecular mechanism of the hepatoprotective effect of SPRT.

## 4. Conclusion

A novel superfine particle processing technique was developed for* Radix Tetrastigma*. SPRT can prevent liver cell apoptosis and create long-term clinical application for sustainable* Radix Tetrastigma*. Superfine grinding technology can be used for the processing of other rare herbal medicines.

## Figures and Tables

**Table 1:** Effects of SPRT on bax and caspase-3 expression in rat liver treated with CCl_4_.

Group	Concentration	Mean density	Mean density
		Bax	Caspase-3
Control	—	0	0
Model	—	0.0683 ± 0.0161^##^	0.0536 ± 0.0188^##^
Bifendate	200 mg/kg	0.0539 ± 0.0192	0.0394 ± 0.0154
SPRT-1	0.3 g/kg	0.0624 ± 0.0170	0.0388 ± 0.0203
SPRT-2	0.6 g/kg	0.0450 ± 0.0179*	0.0358 ± 0.0148*
SPRT-3	1.2 g/kg	0.0359 ± 0.0245**	0.0311 ± 0.0064*

Data are expressed as mean ± SD (*n* = 10). ^##^ *P* < 0.01 compared to normal controls; **P < 0.01, *P < 0.05 compared to disease model group.
